# Risks of asphyxia-related neonatal complications in offspring of mothers with type 1 or type 2 diabetes: the impact of maternal overweight and obesity

**DOI:** 10.1007/s00125-017-4279-2

**Published:** 2017-04-13

**Authors:** Sven Cnattingius, Anna Lindam, Martina Persson

**Affiliations:** Clinical Epidemiology Unit (T2), Department of Medicine Solna, Karolinska Institutet, SE-171 76 Stockholm, Sweden

**Keywords:** Apgar score, Asphyxia, Asphyxia-related neonatal morbidity, Body mass index (BMI), Diabetes type 1, Diabetes type 2, Obesity, Overweight

## Abstract

**Aims/hypothesis:**

We aimed to compare the risks of severe asphyxia-related neonatal complications in the offspring of mothers with type 1 or type 2 diabetes, and to assess the impact of maternal overweight/obesity on these risks.

**Methods:**

This was a population-based study of 1,343,751 live-born singleton infants in Sweden between 1997 and 2011, including 5941 and 711 infants of mothers with type 1 and type 2 diabetes, respectively. ORs with 95% CIs were calculated for low Apgar score (0–6) at 5 min after birth, hypoxic ischaemic encephalopathy and neonatal seizures.

**Results:**

The rates of a low Apgar score were 0.9%, 2.6% and 2.1% in the offspring of mothers without diabetes or with type 1 or type 2 diabetes, respectively. After controlling for maternal confounders (including BMI), the risk of a low Apgar score increased in the offspring of mothers with type 1 diabetes (OR 2.67, 95% CI 2.23, 3.20) but not in the offspring of mothers with type 2 diabetes (OR 1.25, 95% CI 0.66, 2.35). The ORs of hypoxic ischaemic encephalopathy or neonatal seizures were increased in the offspring of mothers with type 1 diabetes (OR 3.41, 95% CI 2.58, 4.49) and type 2 diabetes (OR 2.54, 95% CI 1.13, 5.69). Maternal overweight/obesity was a risk factor for asphyxia-related neonatal complications and low Apgar scores in the offspring of mothers with type 1 diabetes and mothers without diabetes.

**Conclusions/interpretation:**

The risks of a low Apgar score and severe asphyxia-related neonatal complications are increased in the offspring of mothers with type 1 or type 2 diabetes. Maternal overweight/obesity is an important contributing factor.

## Introduction

In spite of advances in the management of pregnant women with type 1 or type 2 diabetes, the risks of neonatal morbidity and mortality remain increased [1, 2]. Low Apgar scores are more frequent in the offspring of mothers with type 1 diabetes [3] and are associated with markedly increased risks of neonatal mortality and morbidity [4, 5]. It is, however, unclear whether the risks of a low Apgar score or other asphyxia-related neonatal complications are also increased in the offspring of mothers with type 2 diabetes.

Maternal hyperglycaemia leads to fetal hyperglycaemia and hyperinsulinaemia. Data from both experimental and human studies support the hypothesis that fetal hyperglycaemia increases the risk of fetal hypoxia [6, 7]. We have previously demonstrated that maternal obesity in early pregnancy increases the risks of low Apgar scores and asphyxia-related neonatal complications in the infants of mothers without diabetes [8]. The prevalence of severe obesity continues to increase in women of reproductive age [9], as it does in women with type 1 or type 2 diabetes [10, 11]. In the offspring of mothers with type 1 diabetes, maternal obesity increases the risks of major malformations and macrosomia [12]. However, it is unclear whether maternal overweight and obesity also increase the risks of asphyxia-related neonatal complications in the offspring of mothers with type 1 diabetes.

In this study, we investigated the offspring of mothers with type 1 or type 2 diabetes with respect to the risks of a low Apgar score (0–6) at 5 min and asphyxia-related neonatal morbidity (neonatal seizures and hypoxic ischaemic encephalopathy [HIE]). In addition, we investigated the impact of overweight and obesity on these risks in the offspring of mothers with type 1 diabetes and without diabetes.

## Methods

### Data sources

This study was based on prospectively collected data from nationwide Swedish registries. The individual record linkage between registries was done by using the unique personal identification number assigned to all residents in Sweden [13]. The Medical Birth Register (MBR) contains data on more than 98% of all births in Sweden since 1973. The MBR is continuously checked for quality by the National Board of Health and Welfare, and the validity of most variables is considered to be high [14]. Using standardised forms, physicians and midwives prospectively collect information during pregnancy, delivery and the neonatal period. Maternal and neonatal diagnoses are classified according to the ICD, and since 1997 the ICD-10 (www.who.int/classifications/icd/en/) has been used. Infants with diagnoses of neonatal seizures and/or HIE were identified based on ICD-10 codes in the MBR and/or in the National Patient Register for inpatient care [15]. Information on the mother’s country of birth and level of education was retrieved from the Total Population Register and Education Register, respectively. The study was approved by the Research and Ethics Committee at Karolinska Institutet (No. 2012/1813-31/4).

### Study population

Between 1997 and 2011, the MBR included information on 1,441,623 live singleton births. We excluded infants with malformations (*n* = 84,711, ICD 10 codes Q00-Q99), offspring of mothers with an unclear diagnosis of diabetes (*n* = 64) and offspring of mothers with gestational diabetes (*n* = 13,097). Thus, the final study population included 1,343,751 live singleton births.

### Exposures

The main exposures were maternal pregestational diabetes and maternal BMI. Mothers with type 1 or type 2 diabetes were identified based on ICD-10 codes O240 and O241, respectively. The reference group included 1,337,099 offspring of mothers without diabetes. Maternal BMI (kg/m^2^) was based on measured weight and self-reported height at the first antenatal visit, which usually took place in the first trimester [16]. Women were categorised as underweight (BMI <18.5 kg/m^2^), normal weight (BMI 18.5 to <25 kg/m^2^), overweight (BMI 25 to <30 kg/m^2^) or obese (BMI ≥30 kg/m^2^) according to the WHO criteria [17].

### Covariates

The overall analyses included covariates of maternal age, height, BMI, self-reported smoking status, level of education, parity, mother’s country of birth and year of delivery. These variables were categorised as shown in Table 1.

**Table 1:** Maternal and infant characteristics for pregnancies in mothers with type 1 or type 2 diabetes and in women without diabetes

Characteristic	Diabetes		No diabetes
	Type 1	Type 2	*n* (%)
	*n* (%)	*n* (%)	
Maternal age (years)			
≤24	767 (12.9)	35 (4.9)	203,418 (15.2)
25–29	1807 (30.4)	119 (16.7)	415,759 (31.1)
30–34	2062 (34.7)	221 (31.1)	459,539 (34.4)
≥35	1292 (21.7)	334 (47.0)	255,232 (19.1)
Missing	13 (0.2)	2 (0.3)	3151 (0.2)
Height (cm)			
≤164	2034 (34.2)	318 (44.7)	479,637 (35.9)
165–174	2856 (48.1)	292 (41.1)	647,159 (48.4)
≥175	576 (9.7)	59 (8.3)	123,677 (9.2)
Missing	475 (8.0)	42 (5.9)	86,626 (6.5)
BMI (kg/m^2^)			
≤24.9	2553 (43.0)	120 (16.9)	758,158 (56.7)
25.0–29.9	1765 (29.7)	164 (23.1)	295,853 (22.1)
30.0–34.9	624 (10.5)	183 (25.7)	91,275 (6.8)
≥35.0	291 (4.9)	171 (24.1)	35,401 (2.6)
Missing	708 (11.9)	73 (10.3)	156,412 (11.7)
Smoking			
No smoking	4876 (82.1)	594 (83.5)	1,146,998 (85.8)
Smoking	608 (10.2)	83 (11.7)	125,273 (9.4)
Missing	457 (7.7)	34 (4.8)	64,828 (4.8)
Education (years)			
≤11	1559 (26.2)	307 (43.2)	331,538 (24.8)
12	1613 (27.2)	162 (22.8)	340,267 (25.4)
≥13	2676 (45.0)	207 (29.1)	631,335 (47.2)
Missing	93 (1.6)	35 (4.9)	33,959 (2.5)
Parity			
First birth	2659 (44.8)	235 (33.1)	591,629 (44.2)
Second or more	3282 (55.2)	476 (66.9)	745,470 (55.8)
Maternal birth country			
Nordic country^a^	5416 (91.7)	427 (61.1)	1,087,461 (82.4)
Non-Nordic country	487 (8.3)	272 (38.9)	232,967 (17.6)
HT			
Chronic HT	251 (4.2)	53 (7.5)	7691 (0.6)
Pre-eclampsia (PE)	845 (14.2)	54 (7.6)	35,554 (2.7)
No HT/PE	4845 (81.6)	604 (85.0)	1,293,854 (96.8)
Delivery			
Caesarean section	3011 (50.7)	303 (42.6)	201,654 (15.1)
Vaginal instrumental	580 (9.8)	41 (5.8)	101,424 (7.6)
Non-instrumental	2350 (39.6)	367 (51.6)	1,034,021 (77.3)
Gestational age (weeks)			
<32	106 (1.8)	14 (2.0)	7570 (0.6)
32–36	1066 (17.9)	78 (11.0)	52,419 (3.9)
≥37	4574 (77.0)	610 (85.8)	1,271,900 (95.1)
Missing	195 (3.3)	9 (1.3)	5210 (0.4)
Birthweight (g)			
<2500	291 (4.9)	39 (5.5)	37,568 (2.8)
2500–3999	3311 (55.7)	457 (64.3)	1,033,231 (77.3)
4000–4499	1457 (24.5)	141 (19.8)	209,887 (15.7)
4500–4999	581 (9.8)	53 (7.5)	44,719 (3.3)
>5000	105 (1.8)	12 (1.7)	6340 (0.5)
Missing	196 (3.3)	9 (1.3)	5354 (0.4)
Birthweight for gestational age (percentiles)			
<3	61 (1.0)	9 (1.3)	20,028 (1.5)
3 to <10	104 (1.8)	23 (3.2)	62,598 (4.7)
10 to 90	2505 (42.2)	418 (58.8)	1,089,066 (81.4)
>90 to 97	1095 (18.4)	111 (15.6)	114,136 (8.5)
>97	1981 (33.3)	141 (19.8)	46,061 (3.4)
Missing	195 (3.3)	9 (1.3)	5210 (0.4)
Year of birth			
1997–2001	1563 (26.3)	135 (19.0)	395,388 (29.6)
2002–2006	2027 (34.1)	187 (26.3)	446,532 (33.4)
2007–2011	2351 (39.6)	389 (54.7)	495,179 (37.0)

^a^Nordic country of birth includes Sweden, Denmark, Finland, Iceland and Norway

HT, hypertension; PE, pulmonary embolism

### Outcomes

We estimated the risks of a low Apgar score (0–6) at 5 min, neonatal seizures (ICD-10 code P90) and HIE (ICD-10 codes P 91.0, 91.3-P91.6, P918 and P919). Owing to limitations of statistical power, neonatal seizures and HIE were analysed as one composite outcome.

### Statistical analysis

The rates of all outcomes were calculated for the offspring of mothers with type 1 or type 2 diabetes and for the infants of mothers without diabetes. ORs with 95% CIs were calculated using logistic regression, with the offspring of mothers without diabetes as the reference. The first adjusted model included information on maternal age, height, smoking status, level of education, parity, maternal country of birth and year of delivery. In a second model, we additionally adjusted for maternal BMI. Height was included in the adjusted model as it is independently associated with emergency Caesarean section [18], which may in turn reflect an increased risk of birth asphyxia-related complications. In stratified analyses, the risks of a low Apgar score and asphyxia-related neonatal morbidity (neonatal seizures or HIE) were investigated in subgroups of infants born to normal-weight, overweight or obese mothers. Owing to limitations of statistical power, stratified analyses were confined to the offspring of mothers with type 1 diabetes and those without diabetes. Interactions between type 1 diabetes (yes/no) and maternal BMI (<25 vs ≥25 kg/m^2^) with risks of low Apgar score and asphyxia-related morbidity were tested for with likelihood tests. Interactions were judged significant at *p* < 0.05. Sensitivity analyses, confined to term infants with a birthweight for gestational age within the 10th–90th percentiles of mothers without hypertensive diseases (chronic hypertension and pre-eclampsia), were also performed. A general estimation equation was applied to account for repeated pregnancies.

## Results

From 1997 through 2011, there was a total of 5941 live singleton births to mothers with type 1 diabetes, 711 to mothers with type 2 diabetes and 1,337,099 to mothers without diabetes. Mothers with type 2 diabetes were more often of non-Nordic origin, older and multiparous, and had a shorter time in education, than mothers with type 1 diabetes and mothers without diabetes. The prevalence of overweight/obesity and smoking was also higher in women with type 2 diabetes (Table 1).

Compared with mothers without diabetes, mothers with type 1 or type 2 diabetes had higher rates of pre-eclampsia, chronic hypertension, Caesarean section and vaginal instrumental delivery. Pre-eclampsia, Caesarean section and vaginal instrumental delivery were most common in mothers with type 1 diabetes, whereas chronic hypertension was most common in mothers with type 2 diabetes. Rates of preterm birth and large-for-gestational age infants (90th–97th percentile and >97th percentile) were also much increased in the offspring of mothers with diabetes, in particular in those of mothers with type 1 diabetes (Table 1).

### Low Apgar score

At 5 min, a low Apgar score (0–6) was recorded in 2.6% (*n* = 153) of the offspring of mothers with type 1 diabetes and in 0.9% of the offspring of non-diabetic mothers, corresponding to an almost threefold increased risk after adjustment for confounders (Table 2). A low Apgar score was recorded in 2.1% (*n* = 15) of the offspring of mothers with type 2 diabetes, corresponding to a more than doubled risk. However, the risk of a low Apgar score in the offspring of mothers with type 2 diabetes was not significant after adjustment for maternal confounders (model 1), and taking maternal BMI into account as well (model 2) further reduced the risk (Table 2).

**Table 2:** Risks of a low Apgar score (0–6) at 5 min and convulsions or HIE in the offspring of women with type 1 or type 2 diabetes or without diabetes

	No Diabetes	Diabetes	
		Type 1	Type 2
Low Apgar score			
Low	11,887 (0.9)	153 (2.6)	15 (2.1)
Not low	1,315,428 (99.1)	5699 (97.4)	691 (97.9)
Crude	Reference	2.99 (2.54, 3.51)	2.28 (1.34, 3.89)
Model 1	Reference	2.92 (2.46, 3.48)	1.60 (0.85, 3.01)
Model 2	Reference	2.67 (2.23, 3.20)	1.25 (0.66, 2.35)
Convulsions or HIE			
Convulsions/HIE	3782 (0.3)	61 (1.0)	9 (1.3)
No convulsions/HIE	1,333,317 (99.7)	5880 (99.0)	702 (98.7)
Crude	Reference	3.56 (2.75, 4.61)	4.59 (2.37, 8.9)
Model 1	Reference	3.68 (2.81, 4.81)	4.31 (2.13, 8.71)
Model 2	Reference	3.40 (2.58, 4.48)	2.54 (1.13, 5.69)

Data are *n* (%) or OR (95% CI)

Risks of low Apgar scores are calculated based on infants with information on Apgar scores

Model 1: adjusted for maternal age, maternal height, smoking, education, parity, maternal country of birth and year of delivery

Model 2: adjusted for BMI, maternal age, maternal height, smoking, education, parity, maternal country of birth and year of delivery

### Asphyxia-related morbidity

Rates of severe asphyxia-related neonatal morbidity (defined as either neonatal seizures and/or HIE) were comparable in the offspring of mothers with type 1 and type 2 diabetes, and were markedly higher than in the offspring of mothers without diabetes. The risk of asphyxia-related morbidity was more than three times increased in the offspring of mothers with type 1 diabetes, and was only slightly reduced after adjustment for confounders. In the offspring of mothers with type 2 diabetes, the corresponding crude risk showed a greater than four times increase, but decreased after adjustment for maternal BMI (Table 2).

### Sensitivity analyses

Confining the analyses to term offspring with a birthweight for gestational age between the 10th and 90th percentiles of those seen in mothers without hypertensive diseases slightly reduced the risk of a low Apgar score in both the offspring of mothers with type 1 and type 2 diabetes (Table 3). Corresponding sensitivity analyses of severe asphyxia-related morbidity revealed even higher risks in the offspring of mothers with type 1 diabetes, whereas the risks were not increased in the offspring of mothers with type 2 diabetes (Table 3).

**Table 3:** Sensitivity analysis for the risk of a low Apgar score and convulsions or HIE for women with type 1 or type 2 diabetes or without diabetes: offspring born at term with birthweight in the 10th–90th percentile to normotensive mothers

	No Diabetes	Diabetes	
		Type 1	Type 2
Low Apgar score			
Low	6781 (0.7)	26 (1.4)	5 (1.5)
Not low	1,010,307 (99.3)	1871 (98.6)	332 (98.5)
Crude	Reference	2.09 (1.42, 3.08)	1.82 (0.68, 4.93)
Model 1	Reference	1.96 (1.31, 2.94)	1.33 (0.42, 4.22)
Model 2	Reference	1.76 (1.15, 2.69)	1.06 (0.33, 3.35)
Convulsions or HIE			
Convulsions/HIE	2434 (0.2)	19 (1.0)	2 (0.6)
No convulsions/HIE	1,021,680 (99.8)	1905 (99.0)	336 (99.4)
Crude	Reference	4.20 (2.67, 6.60)	2.52 (0.62, 10.3)
Model 1	Reference	4.10 (2.57, 6.54)	1.31 (0.17, 9.82)
Model 2	Reference	4.05 (2.54, 6.45)	1.07 (0.14, 8.07)

Data are *n* (%) or OR (95% CI)

Model 1: adjusted for maternal age, maternal height, smoking, education, parity, maternal country of birth and year of delivery

Model 2: adjusted for BMI, maternal age, maternal height, smoking, education, parity, maternal country of birth and year of delivery

### Maternal overweight and obesity and risk of low Apgar score

In the offspring of mothers with type 1 diabetes, the rate of a low Apgar score increased with maternal overweight and obesity (Table 4). Compared with the offspring of normal-weight mothers with type 1 diabetes, the risk of a low Apgar score increased with maternal overweight and obesity among the offspring of mothers with type 1 diabetes. Similar increments in risk associated with maternal overweight and obesity were found in the offspring of mothers without diabetes (Table 4). No significant interactions were found between type 1 diabetes (yes/no) and BMI (<25 or ≥25 kg/m^2^) with respect to the risk of a low Apgar score (*p* = 0.71) and asphyxia-related morbidity (*p* = 0.33). Owing to limited statistical power, it was not possible to perform BMI analyses in the offspring of mothers with type 2 diabetes.

**Table 4:** Maternal BMI and risks of a low Apgar score (0–6) at 5 min and convulsions or HIE in the offspring of mothers with and without type 1 diabetes

	Mothers with type 1 diabetes^a,b^			Mothers with no diabetes^b,c^		
	BMI ≤24.9	BMI 25.0–29.9	BMI ≥30.0	BMI ≤24.9	BMI 25.0–29.9	BMI ≥30.0
Low Apgar score						
Low	48 (1.9)	54 (3.1)	29 (3.2)	5608 (0.7)	2921 (1.0)	1738 (1.4)
Not low	2476 (98.1)	1685 (96.9)	871 (96.8)	747,553 (99.3)	290,989 (99.0)	124,022 (98.6)
Crude	Reference	1.63 (1.09, 2.43)	1.73 (1.08, 2.75)	Reference	1.34 (1.28, 1.40)	1.87 (1.77, 1.97)
Adjusted	Reference	1.54 (1.03, 2.32)	1.82 (1.12, 2.95)	Reference	1.36 (1.30, 1.43)	1.92 (1.82, 2.04)
Convulsions or HIE						
Convulsions/HIE	24 (0.9)	17 (1.0)	13 (1.4)	1816 (0.2)	920 (0.3)	529 (0.4)
No convulsions/HIE	2529 (99.1)	1748 (99.0)	902 (98.6)	756,342 (99.8)	294,933 (99.7)	126,147 (99.6)
Crude	Reference	0.91 (0.47, 1.73)	1.54 (0.78, 3.02)	Reference	1.30 (1.20, 1.41)	1.75 (1.59, 1.93)
Adjusted	Reference	0.86 (0.45, 1.65)	1.59 (0.80, 3.15)	Reference	1.35 (1.24, 1.46)	1.85 (1.68, 2.05)

Data are *n* (%) or OR (95% CI)

Scores were adjusted for maternal age, maternal height, smoking, education, parity, maternal country of birth and time period

^a^ *n* = 5163; 689 observations were excluded due to missing information on BMI in analyses of low Apgar scores

^b^ *n* = 156,412 observations were excluded due to missing information on BMI in analyses of asphyxia-related morbidity

^c^ *n* = 1,172,831; 154,484 observations were excluded due to missing information on BMI in analyses of low Apgar scores

### Maternal overweight and obesity and risk of asphyxia-related outcomes

Compared with the offspring of normal-weight mothers with type 1 diabetes, the rate of asphyxia-related morbidity was higher in the offspring of obese mothers with type 1 diabetes, but risk estimates were not significantly increased (Table 4). In mothers who did not have diabetes, the risk of asphyxia-related neonatal morbidity was significantly increased in the offspring of overweight and obese mothers, with little change after adjustment for confounders (Table 4).

## Discussion

This nationwide study demonstrates that the risks of a low Apgar score and severe asphyxia-related neonatal morbidity are similarly increased in the offspring of mothers with type 1 and type 2 diabetes. In stratified analyses, we found that maternal overweight and obesity were associated with increased risks of a low Apgar score and severe asphyxia-related neonatal morbidity in the offspring of both mothers with type 1 diabetes and without diabetes. In the offspring of mothers with type 2 diabetes, the increased risks of a low Apgar score and severe asphyxia-related neonatal morbidity were partly attributed to their mothers’ increased rates of overweight and obesity.

Study strengths include the population-based design, with more than 1.3 million births, including almost 6000 births to mothers with type 1 diabetes and around 700 births to mothers with type 2 diabetes. Data on exposure and outcomes were collected prospectively, which limits the risks of selection and recall bias. We also had information on a large number of confounders, including maternal BMI. This enabled us not only to investigate whether risks related to maternal type 1 or type 2 diabetes were due to BMI or other confounders, but also to investigate overweight- and obesity-related risks in women with type 1 diabetes. In the present study, BMI was calculated based on self-reported height and measured weight at the first antenatal visit. The average weight gain in the first trimester is approximately 2 kg [19]. Although the use of pre-pregnancy weight may seem preferable, it is by necessity self-reported, and women tend to under-report their weight and over-report their height [20]. However, we cannot exclude the fact that risks of a low Apgar score and asphyxia-related neonatal morbidity associated with BMI may have been slightly over- or underestimated due to errors of measurement of height and weight.

The MBR does not contain data on duration of diabetes, prevalence of pre-existing microangiopathy or glycaemic control during pregnancy or at delivery. Thus, the impact of these variables on the risks of a low Apgar score and asphyxia-related neonatal morbidity could not be assessed. In spite of the large sample size, power was sometimes limited or insufficient, especially for stratified analyses of the offspring of mothers with type 2 diabetes.

To our knowledge, this is the first study to investigate the risks of a low Apgar score and asphyxia-related neonatal morbidity in the offspring of mothers with type 1 or type 2 diabetes. The finding of significantly increased risks in the offspring of mothers with diabetes is of concern, given the severity of these outcomes and the increasing incidence of diabetes [21–24]. Although there are many possible causes of a low Apgar score, low scores are strongly associated with adverse asphyxia-related outcomes. Infants with a low (0–6) Apgar score at 5 min face a 45 times increased risk of neonatal death (death within the first 4 weeks of life) [25], and a 31 times higher risk of cerebral palsy compared with infants with a higher Apgar score [22].

We found comparable risks of low Apgar scores and asphyxia-related neonatal morbidity in the offspring of mothers with type 1 and type 2 diabetes, but the impact of maternal characteristics on the risks differed between the groups. In the offspring of mothers with type 2 diabetes, the risks were largely reduced when maternal age, education, smoking, country of birth and parity were taken into account. In particular, risks were attenuated when also adjusting for maternal BMI. In contrast, in pregnancies in women with type 1 diabetes, the impact of these variables on the risks of a low Apgar score and related outcomes was very limited. Type 1 and type 2 diabetes are characterised by different phenotypes and genetic backgrounds. The duration of disease, level of control, prevalence of angiopathy and other factors of relevance to the risk of birth asphyxia differ substantially between individuals with type 1 and type 2 diabetes.

Type 1 and type 2 diabetes are associated with increased rates of hypertensive disorders in pregnancy, preterm birth, growth restriction and large-for-gestational age infants. These conditions are well-known risk factors for birth asphyxia [26]. When excluding the offspring of hypertensive mothers, preterm infants and infants with a low or high birthweight for gestational age from sensitivity analyses, the risks of a low 5 min Apgar score and asphyxia-related morbidity were largely reduced in pregnancies in mothers with type 2 diabetes. In contrast, in pregnancies where the mother had type 1 diabetes, these exclusions had limited impact on risk estimates. In particular, risks of convulsions and HIE also remained high in term, normal-weight offspring of mothers with type 1 diabetes. These findings suggest that the pathophysiological background for the increased risk of birth asphyxia differs between pregnancies in mothers with type 1 and type 2 diabetes. It must be recalled that a normal size at birth for pregnancies in mothers with type 1 diabetes does not necessarily reflect normality. For instance, maternal diabetic vasculopathy increases the risk of impaired placental circulation and may compensate for the growth-promoting effects of diabetes and result in misleadingly normal fetal growth in the pregnancies of mothers with type 1 diabetes. This may at least partly explain the increased risks of a low Apgar score, convulsions and HIE observed even in the offspring of mothers with type 1 diabetes with a birthweight between the 10th and 90th percentiles. Furthermore, in pregnancies in mothers with type 1 diabetes, the risks of chronic fetal hypoxia and stillbirth increase continuously from around 32 weeks’ gestation toward term [27]. This may be one factor behind the high risks of low Apgar scores and severe asphyxia-related neonatal complications in offspring born at term to women with type 1 diabetes.

Pregnancies complicated by type 1 or type 2 diabetes are characterised by fetal hyperinsulinaemia. The association between fetal insulin levels, measured as C-peptide concentrations in cord blood, and neonatal fat mass is well recognised [5]. Data from both experimental and clinical studies suggest that fetal hyperinsulinaemia increases the risk of chronic fetal hypoxia [6, 7, 28] but may also be a risk factor for acute hypoxia. It has also been suggested that fetal hyperinsulinaemia increases the risk of fetal cardiomyopathy, which is seen in 20–40% of the offspring of mothers with type 1 diabetes [29, 30]. This condition may be present with and without symptoms and appear to be transient at 1 year after birth [31]. However, cardiomyopathy is a common finding in stillbirths to mothers with type 1 diabetes and is a possible contributory cause of ‘unexplained’ fetal death [32].

In pregnancies complicated by type 1 diabetes, there is already evidence of poorer fetal cardiac function in the first trimester, and at 36 weeks of gestation a persistent lower right ventricular performance in combination with structural abnormalities has been reported [33]. Furthermore, the infants of mothers with type 1 diabetes have altered heart variability (rate/rhythm) at birth, with a pattern suggestive of sympathetic predominance [34] and elevated levels of biomarkers of cardiac dysfunction [35]. Against this background, it is possible that alterations in cardiac structure and function, in combination with intrauterine hyperglycaemia and hyperinsulinaemia, lead to increased susceptibility to hypoxia in the fetuses of mothers with type 1 diabetes.

Another possible contributing factor behind the increased risks of asphyxia in the offspring of mothers with type 1 diabetes relates to abnormal placental structure and function. Placentas from pregnancies of mothers with type 1 diabetes are generally larger than normal, and common histological changes include villous immaturity, chorangiosis and the presence of nucleated fetal erythrocytes [36]. These placental abnormalities are associated with chronic hypoxia and are often seen in spite of good glycaemic control [36].

We previously demonstrated that the risk of low Apgar scores increases with maternal obesity in all singleton births [8, 37]. The results of the present study demonstrate a similar pattern in the offspring of mothers with type 1 diabetes. The mechanism behind these findings is unclear. In pregnancies of women with diabetes or obesity, there is a high prevalence of large-for-gestational age infants. Large babies are at risk of traumatic delivery, which may in turn increase risk of birth asphyxia [38]. However, even after excluding large-for-gestational age infants from the analyses, the risks of low Apgar scores and related outcomes in the offspring of mothers with type 1 diabetes remained essentially unchanged.

The Hyperglycemia and Adverse Pregnancy Outcome (HAPO) study found an independent association between maternal overweight/obesity and risk of hyperinsulinaemia in cord blood [39], which is in turn a risk factor for fetal hypoxia. In addition, obesity in pregnancy is associated with a state of insulin resistance, inflammation and endothelial dysfunction in maternal and placental tissues [40, 41]. It is possible that fetal hyperinsulinaemia and placental inflammation and vascular dysfunction contribute to increased risks of birth asphyxia with increasing maternal BMI in pregnancies with and without type 1 diabetes in the mother.

In conclusion, the offspring of mothers with type 1 or type 2 diabetes have increased risks of low Apgar scores and severe asphyxia-related neonatal morbidity. Concomitant diabetes and overweight/obesity are associated with even higher risks. Risk factors for neonatal asphyxia partly differ between mothers with type 1 and type 2 diabetes. The prevalence of conventional risk factors, such as higher maternal age, obesity, smoking and lower educational levels, is higher in women with type 2 diabetes than women with type 1 diabetes. These factors have a greater impact on the risk of low Apgar scores in the offspring of mothers with type 2 diabetes compared with the offspring of mothers with type 1 diabetes. In addition, as demonstrated in the sensitivity analyses, the role of potential fetal mediators (preterm birth and abnormal fetal size) for risk of birth asphyxia is more evident for mothers with type 2 diabetes. We believe that these results are also applicable to women with diabetes in countries similar to Sweden. Women with type 1 or type 2 diabetes should be encouraged to strive towards a normal body weight before pregnancy.

## Abbreviations

HIE: Hypoxic ischaemic encephalopathy
MBR: Medical Birth Register
